# Alternative Roles for CRISPR/Cas Systems in Bacterial Pathogenesis

**DOI:** 10.1371/journal.ppat.1003621

**Published:** 2013-10-17

**Authors:** Timothy R. Sampson, David S. Weiss

**Affiliations:** 1 Department of Microbiology and Immunology, Microbiology and Molecular Genetics Program, Emory University School of Medicine, Atlanta, Georgia, United States of America; 2 Emory Vaccine Center, Emory University, Atlanta, Georgia, United States of America; 3 Yerkes National Primate Research Center, Emory University, Atlanta, Georgia, United States of America; 4 Division of Infectious Diseases, Department of Medicine, Emory University School of Medicine, Atlanta, Georgia, United States of America; University of North Carolina at Chapel Hill School of Medicine, United States of America

## CRISPR/Cas Loci Encode Adaptive, RNA-Directed Nucleic Acid Restriction Systems

CRISPR (clustered regularly interspaced short palindromic repeats)/Cas (CRISPR-associated) systems are highly specific bacterial defenses against foreign genetic elements derived from bacteriophages, plasmids, or extracellular chromosomal DNA [1]. These systems consist of a CRISPR array (crRNA array; composed of unique spacer sequences flanked by short repeats) and adjacently encoded Cas proteins [1]. Following transcription, the crRNA array is processed into individual CRISPR RNAs (crRNA) containing a spacer and a partial repeat [2]. The spacers hybridize to complementary nucleic acid targets, triggering their degradation by Cas proteins [1]. In addition, the Cas proteins Cas1 (a dsDNA endonuclease) and Cas2 (a dsDNA and/or ssRNA endonuclease) function to integrate new spacer sequences into the crRNA array, an adaptation phase that allows bacteria to subsequently target foreign genetic elements containing these sequences [3], [4].

There are three main types of CRISPR/Cas systems. All contain Cas1 and Cas2, but are distinguished by specific Cas proteins involved in crRNA maturation, nucleic acid targeting, and cleavage [1]. Specifically, the Type II CRISPR/Cas systems are associated with pathogenic bacteria including *Neisseria meningitidis*, *Campylobacter jejuni*, and *Streptococcus pyogenes* [1], [5]. These systems require a trans-activating CRISPR RNA (tracrRNA) and an endogenous RNase (RNase III) for maturation of crRNAs, as well as two endonuclease domains within Cas9 for cleavage of each strand of the targeted DNA [2], [6], [7]. In addition to their role in defense against foreign nucleic acid, recent work has demonstrated an alternative functionality of Type II CRISPR/Cas systems as being essential to pathogenesis.

## CRISPR/Cas Systems Play Critical Roles in Pathogenesis

**Figure 1:**
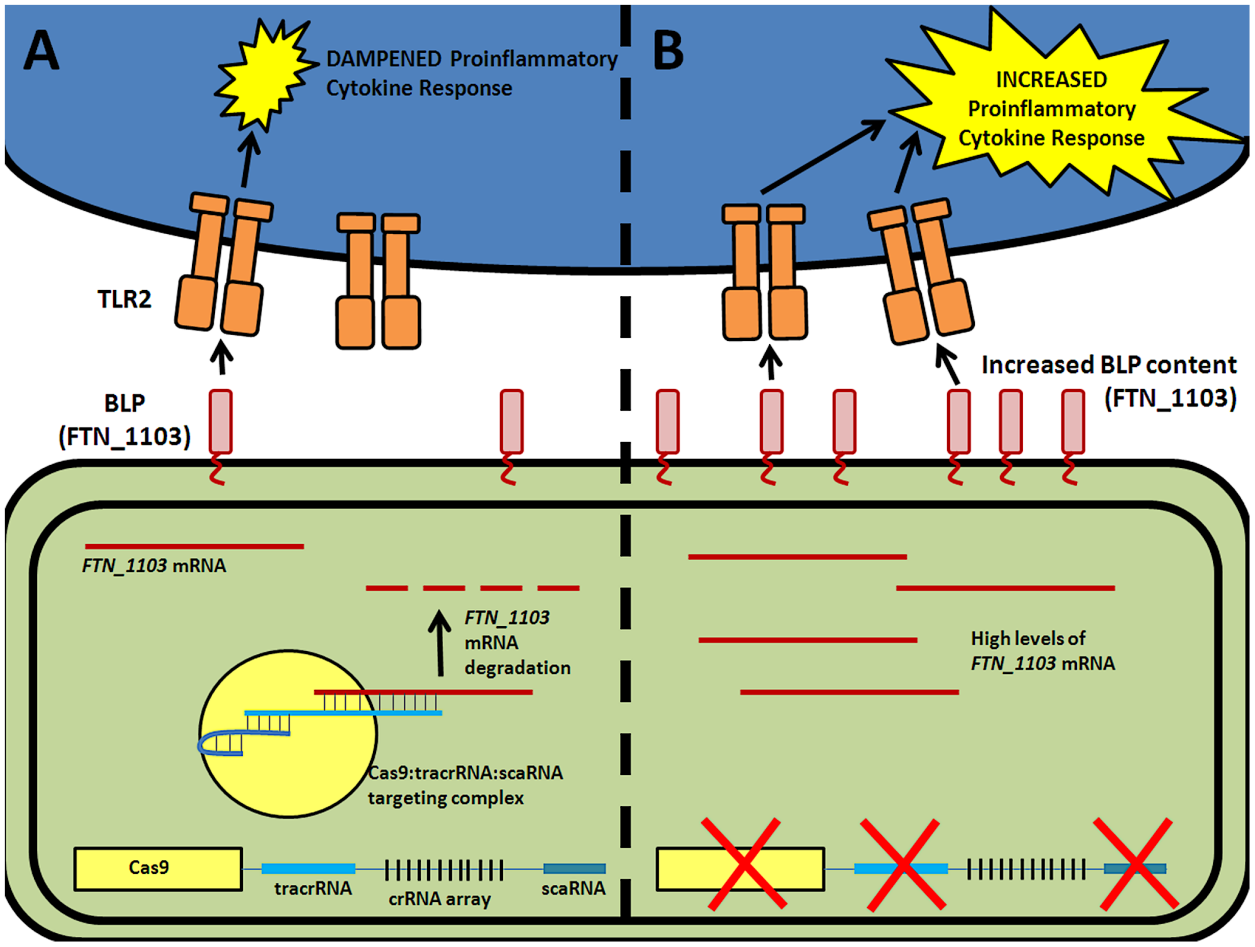
CRISPR/Cas-mediated evasion of innate immune detection by *F. novicida*. (A) Cas9 (yellow), tracrRNA (blue), and scaRNA (teal) act to target the mRNA of a bacterial lipoprotein (red, BLP), ultimately mediating its repression by degradation. Low levels of BLPs result in a dampened TLR2-dependent proinflammatory cytokine response. (B) In the absence of either of these three CRISPR/Cas components, there is an increase in the abundance of BLP mRNA. This results in increased BLP content, and triggers a robust TLR2-dependent proinflammatory cytokine response.

We recently demonstrated that components of the Type II CRISPR/Cas system in *Francisella novicida* are necessary for this intracellular bacterial pathogen to evade detection by a host pattern recognition receptor and cause disease [5]. Cas9, in conjunction with tracrRNA and a novel small RNA termed scaRNA (small, CRISPR/Cas-associated RNA), target an endogenous transcript encoding an immunostimulatory bacterial lipoprotein (BLP), leading to mRNA degradation and decreased transcript levels (Figure 1A) [5]. Surprisingly, this action does not rely on any of the crRNAs, but instead is predicted to utilize tracrRNA to target mRNA. In the absence of this regulation, increased BLP levels trigger the activation of a Toll-like Receptor 2 (TLR2)-dependent proinflammatory response, and result in complete attenuation of the bacteria during infection (Figure 1B). These CRISPR/Cas components are therefore critical to the ability of *F. novicida* to cause disease.

Cas9 is also required for the ability of both *Neisseria meningitidis* and *Campylobacter jejuni* to attach to, invade, and replicate within epithelial cells, traits that are essential to their virulence [5], [8]. Currently, the mechanism of Cas9-mediated pathogenesis by these organisms is unknown. It is likely that Cas9, in conjunction with one or more small RNAs, acts to alter the stability of a transcript and that this is important for virulence, similar to its function in *F. novicida*. Interestingly, the role of Cas9 in *C. jejuni* virulence correlated with strains producing a sialylated lipooligosaccharide structure in the outer envelope [8]. Coupled with the fact that Cas9 regulates production of a membrane BLP in *Francisella*, it is tempting to speculate that CRISPR/Cas systems that control mRNA stability may be widely involved in regulation of envelope structure.

In addition to the role of Cas9 in the virulence of bacterial pathogens, Cas2 has been implicated in the ability of *Legionella pneumophila* to survive within amoebae [9]. Since amoebae are essential to *L. pneumophila* survival in the environment [10], the role of Cas2 in this process may be critical to environmental persistence and subsequent transmission to other hosts. Exactly how Cas2 mediates *Legionella* intracellular survival is unknown. Due to its endonuclease activity involved in CRISPR adaptation, Cas2 is hypothesized to also function in either the processing of small RNA regulators during intracellular infection, or to be a direct mediator of mRNA degradation [9]. Interestingly, Cas9 plays no role in the intracellular survival of *L. pneumophila* in amoebae [9]. Additionally, Cas2 has no currently observed role in *F. novicida* gene regulation or virulence [5], demonstrating that while Type II CRISPR/Cas systems are similar across species, different organisms may have co-opted distinct components for pathogenesis.

## CRISPR/Cas Systems Can Control Bacterial Physiology

Different types of CRISPR/Cas systems have also been observed to contribute to bacterial physiology beyond defense against foreign nucleic acids. The CRISPR/Cas system in *Pseudomonas aeruginosa* plays a role in modulating biofilm formation [11], [12]. While the precise mechanism is unknown, the data suggest that when *P. aeruginosa* is lysogenized by a specific bacteriophage, the CRISPR/Cas system interacts with a particular gene in the chromosomally integrated prophage to inhibit the creation of biofilms [11], [12]. It is unclear if the CRISPR/Cas system targets DNA or mRNA, but it is known that the interaction requires the Cas proteins involved in both crRNA maturation and targeting/degradation, as well as a specific targeting crRNA with sequence similarity to the prophage gene [11], [12]. Given the importance of biofilm formation in the pathogenic life cycle of *P. aeruginosa* [12], it is likely that this intricate CRISPR/Cas regulatory schema plays an important role in infection.

Additionally, Cas1 and the crRNA array in the CRISPR/Cas system of *Escherichia coli* K-12 (also present in EHEC and UPEC strains [1]) play a role in mediating DNA repair [13]. Given the universality of Cas1 in all known CRISPR/Cas systems, it is intriguing to speculate that it may broadly function in this regard. Further, since DNA damage may occur as a product of host defenses during infection (i.e., production of reactive nitrogen and oxygen species) [14], it is interesting to consider that CRISPR/Cas systems may provide pathogens another layer of redundancy in their ability to resist and repair damage incurred during infection.

## Are Self-Targeting CRISPR/Cas Systems Involved in “Autoimmunity” or Gene Regulation?

Another potential example of noncanonical functionality of CRISPR/Cas systems in gene regulation and virulence may involve self-targeting crRNAs with spacer sequences complementary to chromosomally encoded genes [15], [16]. For example, self-targeting crRNAs are predicted to target hypothetical proteins in the pathogens *Clostridium botulinum*, *N. meningitidis*, and *Yersinia pestis*, two sporulation genes and a gene involved in S-layer biosynthesis in *Clostridium tetani*, and the *fdrA* gene involved in protein transport in *Enterobacter* spp. [15]. Since crRNAs are known to target DNA, their specificity for chromosomal genes has been suggested to likely result in detrimental chromosomal cleavage, which can result in large chromosomal deletions, and thus has been termed “autoimmunity” [17]. It has been hypothesized that self-targeting crRNAs can be tolerated if CRISPR/Cas systems in bacteria encoding them are either nonfunctional or are significantly degenerated, thereby preventing “autoimmune” recognition and cleavage of the chromosome [15]. Indeed, CRISPR/Cas systems encoding self-targeting crRNAs often have degenerated Cas proteins [15], [16]. However, this could nonetheless be consistent with a role in gene regulation for at least some self-targeting crRNAs [16]. Inactive Cas1 and Cas2 proteins would not necessarily inhibit self-targeting abilities, but instead prevent acquisition of new crRNAs [3], [4]. Since acquisition of new crRNAs can lead to loss of previously acquired crRNAs [18], degeneration of Cas1 and Cas2 may actually be favored to prevent loss of regulatory crRNAs. Additionally, it has been demonstrated that a catalytically inactive Cas9 is still capable of binding DNA targets and inhibiting transcription, resulting in repression of the targeted gene [19]. Therefore, degenerated Cas proteins could theoretically still participate in gene regulation. Furthermore, the CRISPR/Cas system in *P. aeruginosa*, capable of targeting a chromosomally integrated element without causing chromosomal degradation, is fully functional against bacteriophage infection, suggesting that chromosomal targeting by an active CRISPR/Cas system does not necessarily lead to “autoimmune” events [11], [12], [20]. Finally, as observed in *F. novicida*, if mRNA but not DNA is targeted (i.e., *FTN_1103*) [5], there would be no negative selection against targeting endogenous genes in the chromosome. It is therefore tempting to speculate that self-targeting crRNAs may act as regulatory elements in at least some of the aforementioned and other pathogens.

## Perspectives

While well established to play roles in defending bacteria from bacteriophages and other foreign genetic elements, the critical roles that CRISPR/Cas systems play in the ability of pathogenic organisms to evade host defenses and replicate within the host are just now being appreciated. Given that CRISPR/Cas systems are widely distributed among prokaryotes (∼50% of bacteria and 99% of Archaea) and are present in both pathogenic and commensal organisms [1], as well as their specificity and adaptability, it is very likely that more examples of their alternative functions in gene regulation controlling virulence, commensalism, and broader physiology will be revealed. Future work elucidating how CRISPR/Cas systems contribute to bacterial virulence will allow for the identification of novel host defense evasion strategies that bacterial pathogens utilize during infection.
